# Genome-wide identification and comparative analysis of *CCCH*-type zinc finger genes in diploid and tetraploid cotton (*Gossypium*) species

**DOI:** 10.3389/fpls.2025.1694549

**Published:** 2025-11-18

**Authors:** Gaofei Sun, Panhong Dai, Xinquan Tian, Renhai Peng, Boshen Li, Lei Ma, Xiaomeng Zhang

**Affiliations:** 1 Anyang Institute of Technology, Anyang, China; 2 State Key Laboratory of Cotton Bio-breeding and Integrated Utilization, Institute of Cotton Research, Chinese Academy of Agricultural Sciences, Anyang, China; 3 Faculty of Engineer, The University of Sydney, Sydney, NSW, Australia

**Keywords:** CCCH-type zinc finger proteins, collinearity analysis, expression profiling, functional diversification, abiotic stress

## Abstract

**Introduction:**

*CCCH*-type zinc finger proteins are important transcriptional regulators involved in plant growth, development, and responses to abiotic stress. Despite their significance, a comprehensive characterization of *CCCH* genes in cotton is lacking.

**Methods:**

We systematically identified *CCCH* genes in four cotton species (*Gossypium arboreum*, *G. raimondii*, *G. hirsutum*, and *G. barbadense*) and performed phylogenetic classification, gene structure, conserved motif, and physicochemical property analyses. Collinearity analyses were conducted to assess gene expansion. Promoter regions were examined for hormone- and stress-responsive cis-elements, and expression profiles were analyzed across tissues, developmental stages, and under abiotic stress conditions.

**Results:**

A total of 183 *CCCH* genes were identified and grouped into eight phylogenetic clusters. Comparative analyses revealed both evolutionary conservation and lineage-specific diversification. Gene expansion in tetraploid cotton mainly arose from polyploidization, with most genes retained from diploid progenitors, whereas *GhCCCH21* and *GhCCCH47* were specific to *G. hirsutum*. Promoter analysis uncovered numerous hormone- and stress-responsive elements, including ABRE, CGTCA-motif, and LTR. Tissue-specific expression patterns showed that *GhCCCH24* and *GhCCCH14* are preferentially expressed in ovules and fibers, respectively, while *GhCCCH23*, *GhCCCH51*, and *GhCCCH55* are strongly induced by abiotic stress.

**Discussion:**

These findings reveal the functional diversification of *CCCH* genes in cotton and identify promising candidates for improving stress tolerance and fiber quality, providing a foundation for future functional studies.

## Introduction

Cotton is one of the world’s most important economic crops, supplying natural fibers to the textile industry and serving as a valuable oilseed crop (Zhang et al., 2016). However, throughout its growth, cotton is exposed to various abiotic stress factors, including drought, salinity, and extreme temperatures, all of which have a significant impact on its yield and quality (Mu et al., 2019; Zhu et al., 2024; Esmaeili et al., 2021; Ge et al., 2022). In recent years, the availability of high-quality genome assemblies for both diploid and tetraploid cotton species has facilitated in-depth studies of the molecular mechanisms underlying stress responses (Ge et al., 2022; Mu et al., 2019; Zhu et al., 2024). Notably, the expression of *CCCH* genes has been reported to influence the salt tolerance of cotton plants, suggesting that this gene family plays an important role in stress adaptation (Zhang et al., 2023).

In plants, drought, salinity, and temperature extremes are among the major environmental factors that adversely affect growth, development, and productivity (Kim et al., 2024; Wang et al., 2023a; Balfagón et al., 2020). To cope with these challenges, plants have evolved complex molecular networks in which transcriptional regulation is central to stress adaptation (Kidokoro et al., 2022; Lu et al., 2023; Wang et al., 2023b; Pratx et al., 2024);. Among the diverse transcriptional regulators, zinc finger proteins are particularly important because of their roles in controlling plant development and mediating responses to environmental cues (Wei et al., 2023; Tian et al., 2024; Zhang et al., 2023). CCCH-type zinc finger proteins, characterized by the conserved Cys_3_-His motif, represent a distinct subclass of zinc finger proteins that are widely distributed in both animals and plants (De et al., 1999; DuBois et al., 1990; Deng et al., 2023). Unlike other zinc finger families, CCCH-type proteins have attracted attention for their potential roles in RNA binding and RNA metabolism (Gosztyla et al., 2024). Functional studies in multiple species have shown that *CCCH* genes are involved in stress tolerance. For instance, overexpression of the cotton gene *GhZFP1* in tobacco significantly enhanced the salt tolerance of transgenic plants (Guo et al., 2009), while overexpression of *PeC3H74* increased drought and salt tolerance in *Arabidopsis* and rice (Lan et al., 2023). Similarly, the knockout of *OsTZF1* promotes seed germination and accelerates leaf senescence in rice, while its overexpression enhances tolerance to salt and drought stress by regulating RNA metabolism (Jan et al., 2013). The CCCH protein CaC3H14 in pepper enhances the plant’s resistance to *Ralstonia solanacearum* infection (Qiu et al., 2018). IbC3H18 improves sweet potato tolerance to multiple abiotic stresses by acting as a transcriptional activator (Zhang et al., 2019). In cotton, GhC3H20 has been shown to interact with ABA signaling components GhPP2CA and GhHAB1, thereby enhancing salt tolerance (Zhang et al., 2023). Moreover, genome-wide identification of *CCCH* gene families has been conducted in plants such as *Arabidopsis* (Wang et al., 2008), soybean (Hu and Zuo, 2021), pepper (Tang et al., 2023) and rice (Wang et al., 2025).

In cotton, several functional studies and preliminary analyses have revealed the involvement of CCCH proteins in abiotic stress responses (Guo et al., 2009; Zhang et al., 2023). However, a systematic and comparative investigation of the *CCCH* gene family across diploid and tetraploid cotton species remains limited. Recognizing these earlier contributions, our study seeks to build upon them by addressing unresolved questions regarding their expansion, functional divergence, and potential regulatory roles in stress adaptation. Meanwhile, the availability of high-quality cotton genome assemblies and successful genome-wide investigations of other gene families—including GLK transcription factors (Tang et al., 2024), *FORMIN* genes (Paul et al., 2025), and the *DUF789* family (Hamid et al., 2025)—provide both the rationale and technical feasibility for such an analysis in cotton.

Therefore, we specifically aimed to test the hypothesis that the *CCCH* gene family in cotton has undergone gene family expansion and functional diversification, and that certain members are transcriptionally responsive to abiotic stresses. To this end, we performed genome-wide identification and characterization of *CCCH* genes in diploid and tetraploid cotton species, analyzed their gene structures, chromosomal distributions, conserved motifs, and evolutionary relationships, and examined their expression patterns across tissues and under abiotic stress conditions. This work provides a valuable genomic resource and identifies candidate *CCCH* genes for future functional studies and the genetic improvement of stress tolerance in cotton.

## Materials and methods

### Plant materials, growth conditions, and salt stress treatment

Upland cotton Zhong J0102 (ZJ0102) seedlings were grown in a greenhouse under controlled conditions (24°C, 70-75% relative humidity, 14 h light/10 h dark photoperiod) for three weeks. At the three-leaf stage, plants were irrigated with either water (control) or 0.4% NaCl solution (4 g NaCl per 1000 g sand). True leaves were collected at 0.5, 3, 12, 24, and 48 h after treatment, with three biological replicates per time point. Samples were immediately frozen in liquid nitrogen and stored at -80°C for RNA extraction.

### Identification and characterization of *CCCH* zinc finger family members in cotton

The diploid (*G. arboretum* and *G. raimondii*) and tetraploid (*G. barbadense* and *G. hirsutum*) genome and protein sequence files were downloaded from CottonGen (https://www.cottongen.org/). The *Arabidopsis* genome file was downloaded from Phytozome database. The amino acid sequences of the *Arabidopsis* CCCH (PF00642), as previously reported, were obtained from the Pfam database. PF00642 was then used as a query sequence to identify candidate CCCH protein sequences in four cotton protein sequence files, using HMMER with an E-value threshold of < 1e-5 (http://hmmer.org/). The conserved domain of CCCH in the candidate sequences were confirmed by the SMART (http://smart.emblheidelberg.de/), Pfam (http://pfam.xfam.org/), and NCBI-CDD databases. Biophysical characteristics of the CCCH proteins were obtained from CottonFGD.

### Chromosome mapping and collinearity analysis

Detailed chromosomal mapping of all identified *CCCH* genes in cotton was obtained from GFF genomic files downloaded from the CottonGen database and the chromosomal distribution of these *CCCH* genes was visualized in TBtools (Chen et al., 2020a). To investigate the collinearity among the four cotton species and analyze their syntenic relationships, the complete genome sequences and corresponding genome annotation files of these cotton species were used. The MCScanX tool (Wang et al., 2012) was employed to identify collinear blocks in these genomes. The collinear and homologous chromosomal regions between the A and D genomes of cotton species were visualized separately using multiple synteny plots in TBtools (Chen et al., 2020a).

### Phylogenetic tree, gene structure and conserved motif analysis of cotton *CCCH* genes

The amino acid sequences of all the identified *CCCH* genes from *A. thaliana* and the four species of cotton were downloaded from TAIR (https://www.arabidopsis.org/) and CottonGen (https://www.cottongen.org/). Multiple protein sequences of the CCCHs were aligned using ClustalW program in the Molecular Evolutionary Genetics Analysis (MEGA) with default parameters, and the phylogenetic tree was constructed using the neighbor-joining method with 1,000 bootstrap replicates. The genome sequences and coding sequences of *CCCH* genes were downloaded from CottonGen and the structure of the *CCCH* genes was visualized using TBtools (Chen et al., 2020a). The conserved motifs of *CCCH* genes were identified by MEME.

### Identification of *cis*-regulatory elements of *CCCH* genes

Following common practice in plant promoter analyses, the 2,000 bp sequences upstream of the start codon of each *CCCH* gene were extracted to investigate *cis*-regulatory elements in the promoter regions. These elements were predicted using PlantCARE (http://bioinformatics.psb.ugent.be/webtools/plantcare/html/).

### Expression profiles analysis of *CCCH* genes

The expression profiles (FPKMs) of *CCCH* genes in different tissues (root, stem, leaf, pistil, torus, bract, sepal, petal, filament, and anther), at different developmental processes of fibers (10, 15, 20 and 25 dpa) and ovules (-3, 0, 1, 3, 5, 10, 20, and 25 dpa), and under different abiotic stress conditions (heat, cold, drought and salt treatments) were downloaded from CottonFGD. For each sample, the expression level was represented by the mean value of three biological replicates. Subsequently, the *CCCH* genes were filtered, and only those with a mean FPKM greater than 1 in at least one condition were used to generate the heatmap using R software (R Core Team, 2019).

### Quantitative real-time PCR analysis (qRT-PCR)

To investigate the effects of salt stress on gene expression, qRT-PCR analysis was performed on six *CCCH* genes in the cotton cultivar ZJ0102 at 0.5, 3, 12, 24, and 48 h after treatment. Total RNA was extracted using the RNAprep Pure Plant Kit (DP441, Tiangen, China), and first-strand cDNA was synthesized in a 20 μL reaction using the PrimeScript™ RT Reagent Kit with gDNA Eraser (RR047A, TaKaRa, Japan) according to the manufacturer’s instructions. Quantitative real-time PCR was conducted on a Roche LightCycler 480 II system. Three independent biological replicates were included for each sample. Primer sequences are provided in **Supplementary Table 5**.

## Results

### Identification and phylogenetic analysis of *CCCH* genes in the four cotton species

**Figure 1:**
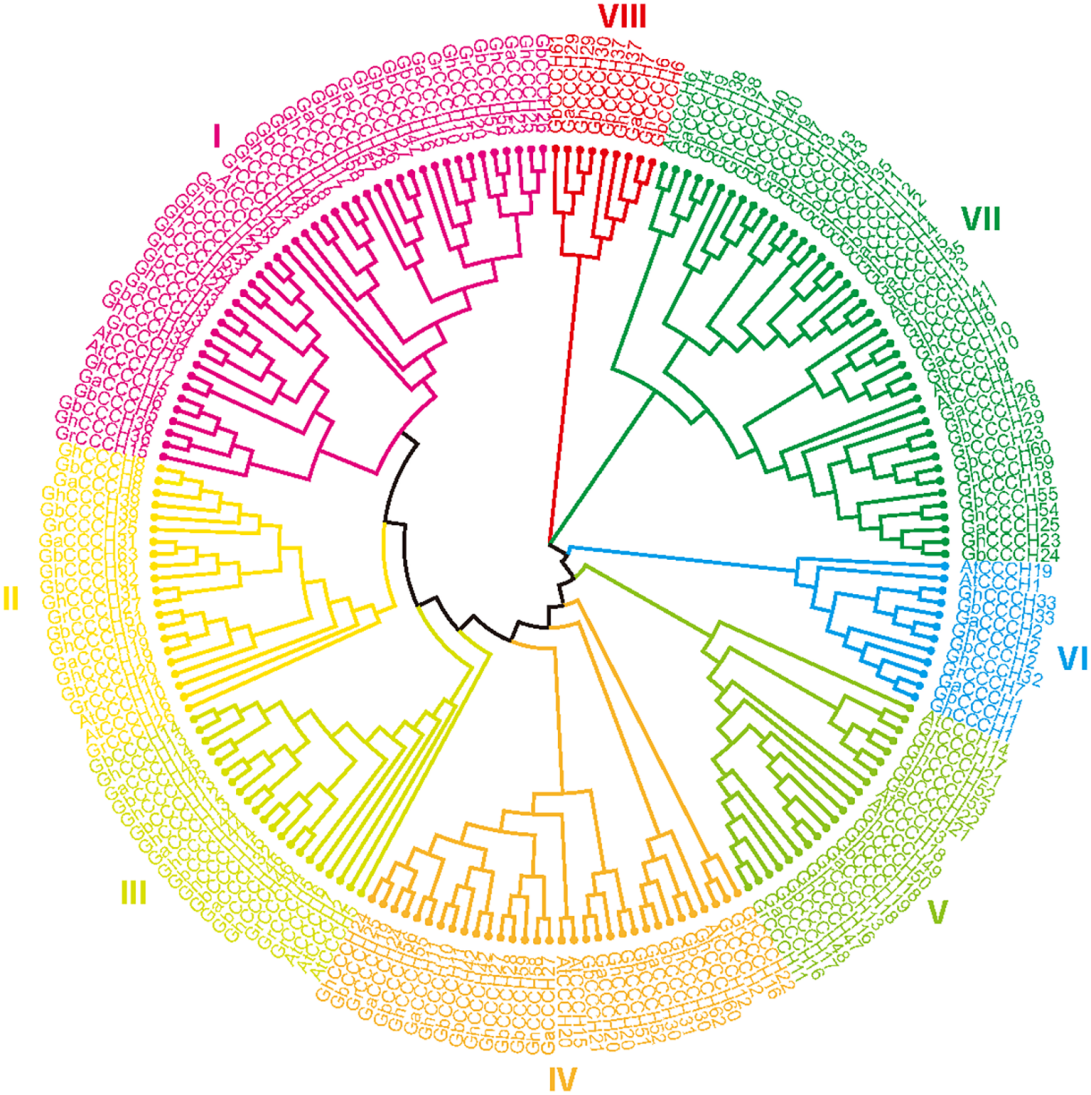
Phylogenetic tree of *CCCH* gene family among *G. arboreum*, *G. raimondii*, *G. barbadense*, *G. hirsutum* and *A. thaliana*. The *CCCH* genes are classified into eight subgroups (I-VIII), each highlighted in a different color.

A total of 183 *CCCH* genes were identified from four cotton species, including 32 genes from *G. arboretum*, 27 genes from *G. raimondii*, 61 genes from *G. hirsutum*, and 63 genes from *G. barbadense*. By performing a multiple sequence alignment of these identified cotton *CCCH* genes with 20 *CCCH* genes from *Arabidopsis*, a phylogenetic tree of the full-length protein sequences was constructed. The phylogenetic analysis divided the 183 *CCCH* genes into eight subgroups, with each subgroup represented by a distinct color (**Figure 1**). The two tetraploid cotton species cluster at the same branch tip, adjacent to one of the diploid species (*G. arboretum* or *G. raimondii*), confirming the closer genetic relationship among tetraploid species within the *CCCH* gene family, in agreement with the established history of cotton domestication.

Additionally, the physicochemical properties of the identified *CCCH* genes in cotton were analyzed (**Supplementary Tables 1**-**4**). The amino acid lengths of these cotton CCCH proteins ranged from 281 to 844 in *G. arboretum*, 279 to 509 in *G. raimondii*, 280 to 530 in *G. hirsutum*, and 225 to 531 in *G. barbadense*. The isoelectric points ranged from 4.938 to 9.924 in *G. arboretum*, 5.156 to 10.075 in *G. raimondii*, 4.878 to 10.369 in *G. hirsutum*, and 4.829 to 10.075 in *G. barbadense*. The molecular weights ranged from 30.623 to 92.279 kDa in *G. arboretum*, 30.833 to 55.78 kDa in *G. raimondii*, 29.692 to 56.625 kDa in *G. hirsutum*, and 23.292 to 56.887 kDa in *G. barbadense*.

### Chromosomal locations of *CCCH* genes in the four cotton species

**Figure 2:**
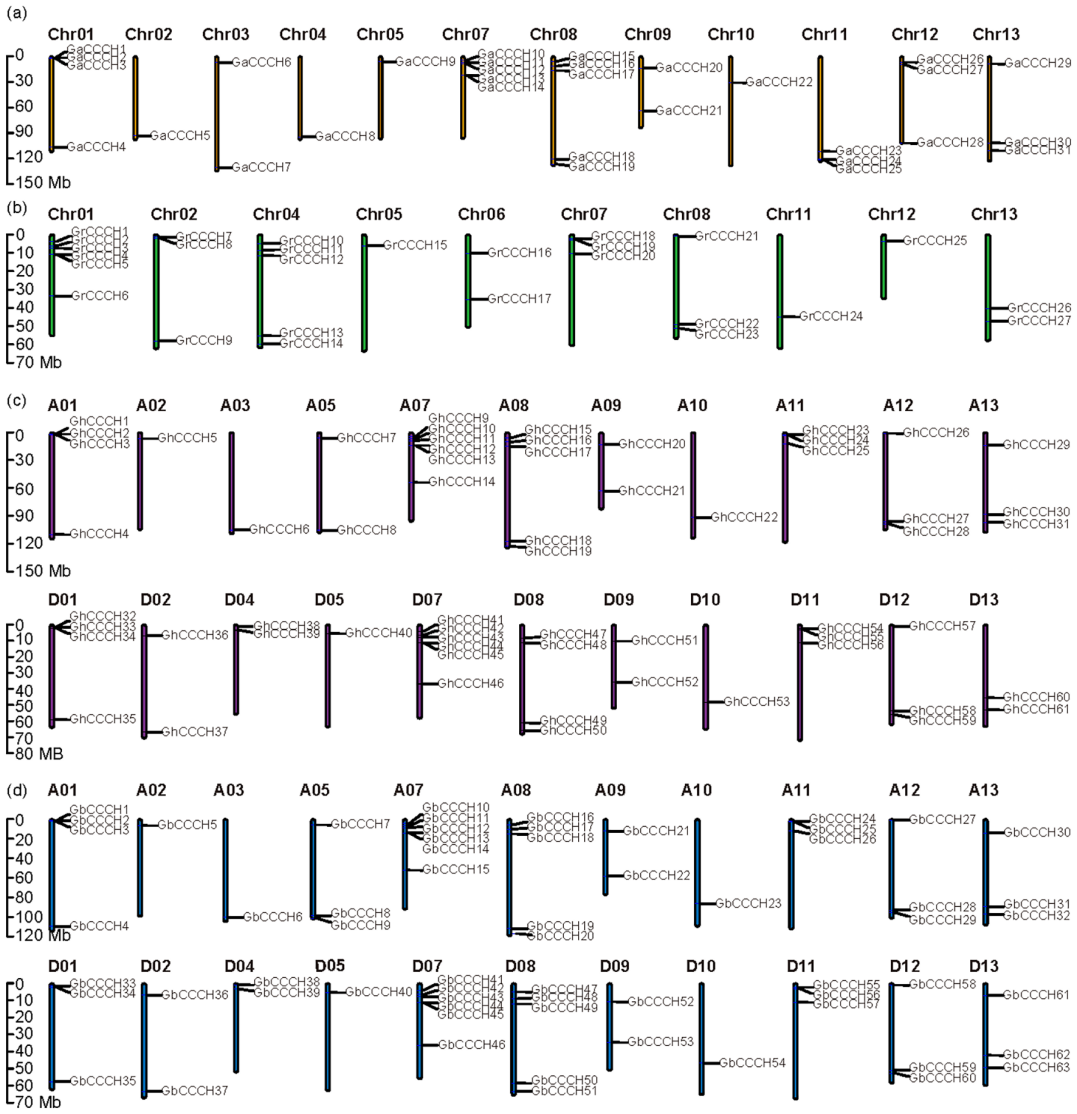
Chromosome distribution of *CCCH* genes in *G*. *arboreum***(a)**, *G*. *raimondii***(b)**, *G*. *hirsutum***(c)** and *G*. *barbadense***(d)**.

**Figure 3:**
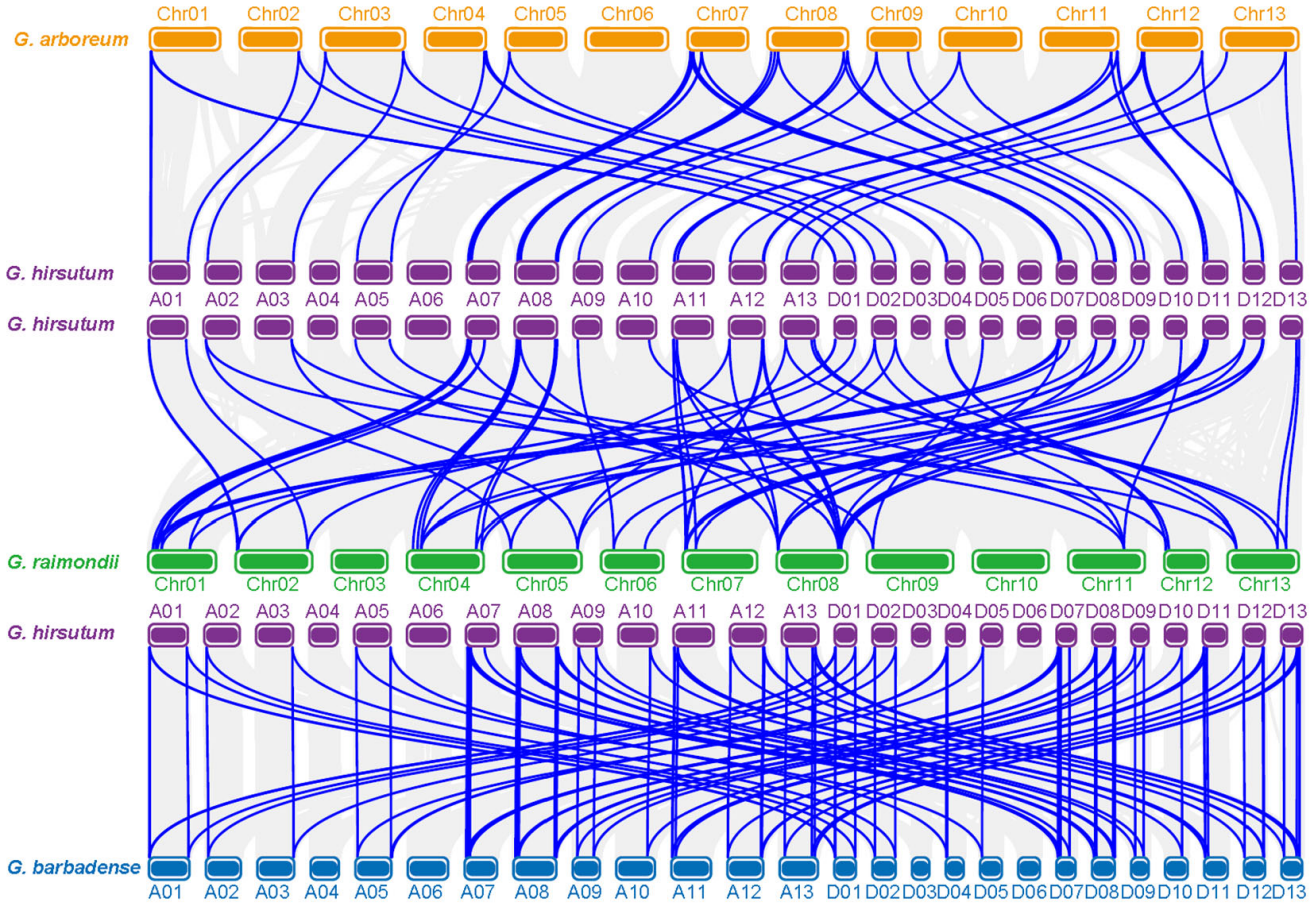
Collinear analysis of *CCCH* genes among four *Gossypium* species (*G. hirsutum*, *G. barbadense*, *G. arboreum*, and *G. raimondii*). The upper and lower panels depict collinear relationships between *G. hirsutum* and *G. arboreum*, *G. raimondii*, and *G. barbadense*, respectively. Gray background lines represent whole-genome synteny, while blue lines highlight syntenic *CCCH* gene pairs.

Based on the genomic and annotation data for diploid and tetraploid cotton, the locations of the identified *CCCH* genes across chromosomes were displayed in detail (**Figure 2**). During domestication, cotton underwent a transition from diploid to tetraploid, resulting in a significant expansion of homologous genes. Consequently, compared to the diploids (*G. arboretum* and *G. raimondii*), both the number of chromosomes and the number of *CCCH* genes were doubled in the tetraploid species (*G. hirsutum* and *G. barbadense*). Due to various genomic mutations (such as deletions, duplications, and insertions), the positions of these genes on the chromosomes have also undergone some changes. Specifically, in *G. arboretum*, *CCCH* genes were located on 12 chromosomes, excluding chromosome Chr06; in *G. raimondii*, they were found on 10 chromosomes, excluding chromosomes Chr03, Chr09, and Chr10; and in both *G. hirsutum* and *G. barbadense*, *CCCH* genes were present on 11 chromosomes of the A subgenome (excluding A04 and A06) and 11 chromosomes of the D subgenome (excluding D03 and D06). The absence of certain chromosomes does not necessarily indicate gene loss. For example, *CCCH* genes located on Chr04 and Chr06 in the diploids have homologous counterparts on A05, A08, A09, and the D subgenome in *G. hirsutum* (**Figure 3**). This suggests that the missing chromosomal locations are likely the result of homeologous gene rearrangements during polyploidization.

It was evident that the localization of *CCCH* genes on the chromosomes was largely consistent between the tetraploid subgenomes (**Figure 2**). For example, four *CCCH* genes were found on both the A01 and D01 chromosomes, with three positioned at the chromosome’s beginning and one at its end. Furthermore, the distribution of *CCCH* genes on chromosomes within the same subgenome was similar between *G. hirsutum* and *G. barbadense*. There were differences in the distribution of *CCCH* genes on the same chromosomes between diploid and tetraploid species, likely reflecting genomic variations that occurred during chromosome duplication. In particular, the highest number of *CCCH* genes was located on chromosomes Chr07 and Chr08 in *G. arboretum*—a feature that has been retained in both *G. hirsutum* and *G. barbadense*, and the distribution patterns of these genes on these two chromosomes were largely consistent across all these three cotton species.

### Collinearity analysis of *CCCH* genes

We conducted pairwise genome-wide collinearity analyses among *Gossypium hirsutum*, *G. arboreum*, *G. raimondii*, and *G. barbadense*, and systematically identified all homologous genes across the four genomes (**Figure 3**). The results revealed clear gene duplication events from the A-genome of *G. arboreum* to both the At and Dt subgenomes of the tetraploid cotton species. A similar pattern of gene expansion and conservation was also observed between the D-genome of *G. raimondii* and the At/Dt subgenomes of *G. hirsutum*.

Among the 61 *CCCH*-type zinc finger genes identified in *G. hirsutum*, 51 were found to have homologous counterparts in both *G. arboreum* and *G. raimondii*, indicating a high degree of conservation of the *CCCH* gene family during cotton evolution. Specifically, 26 of the 31 *CCCH* genes located in the At subgenome of *G. hirsutum* exhibited homology to genes in both diploid progenitors. Similarly, 25 of the 30 *CCCH* genes in the Dt subgenome also had homologs in *G. arboreum* and *G. raimondii*.

In addition to the diploid genomes, a comparative analysis with *G. barbadense* revealed that 59 of the 61 *G. hirsutum CCCH* genes were also conserved in this closely related tetraploid species. However, two genes, *GhCCCH21* and *GhCCCH47*, were uniquely present in *G. hirsutum* but absent from *G. barbadense*, *G. arboreum*, and *G. raimondii*. This absence across both diploid progenitors and a sister tetraploid suggests that these genes may have originated through lineage-specific duplication or neofunctionalization following the polyploidization event that gave rise to *G. hirsutum*. These genes may represent recent evolutionary innovations, and their functional roles in *G. hirsutum* warrant further investigation.

### Structural characterizations and conserved motif analyses of *CCCH* genes

**Figure 4:**
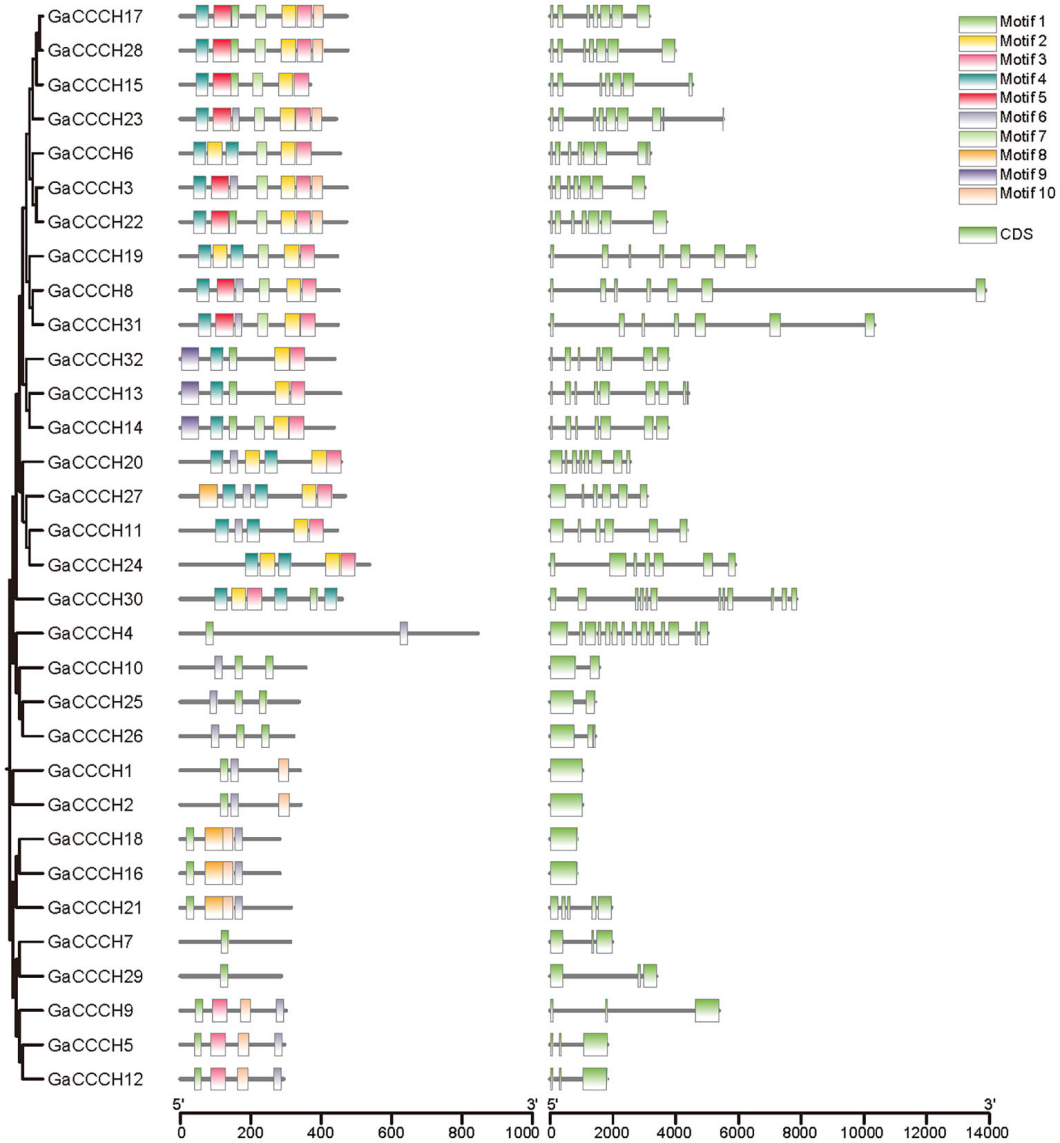
Phylogenetic relationship, motif and gene structures of *CCCHs* in *G. arboreum*. From left to right are evolutionary relationship, structure of conserved motifs and gene architecture.

**Figure 5:**
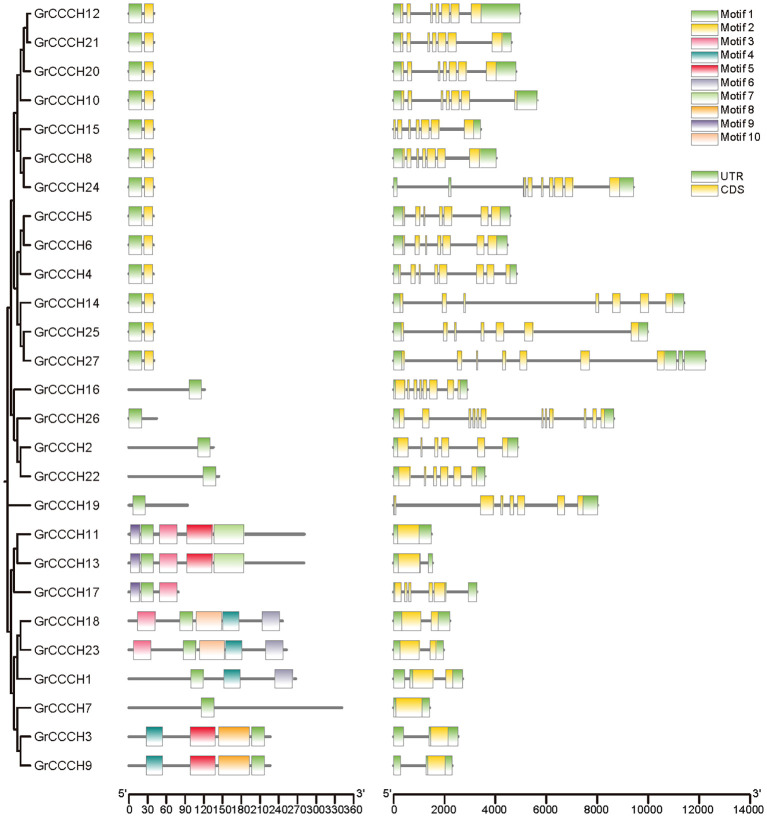
Phylogenetic relationship, motif and gene structures of *CCCHs* in *G. raimondii*. From left to right are evolutionary relationship, structure of conserved motifs and gene architecture.

**Figure 6:**
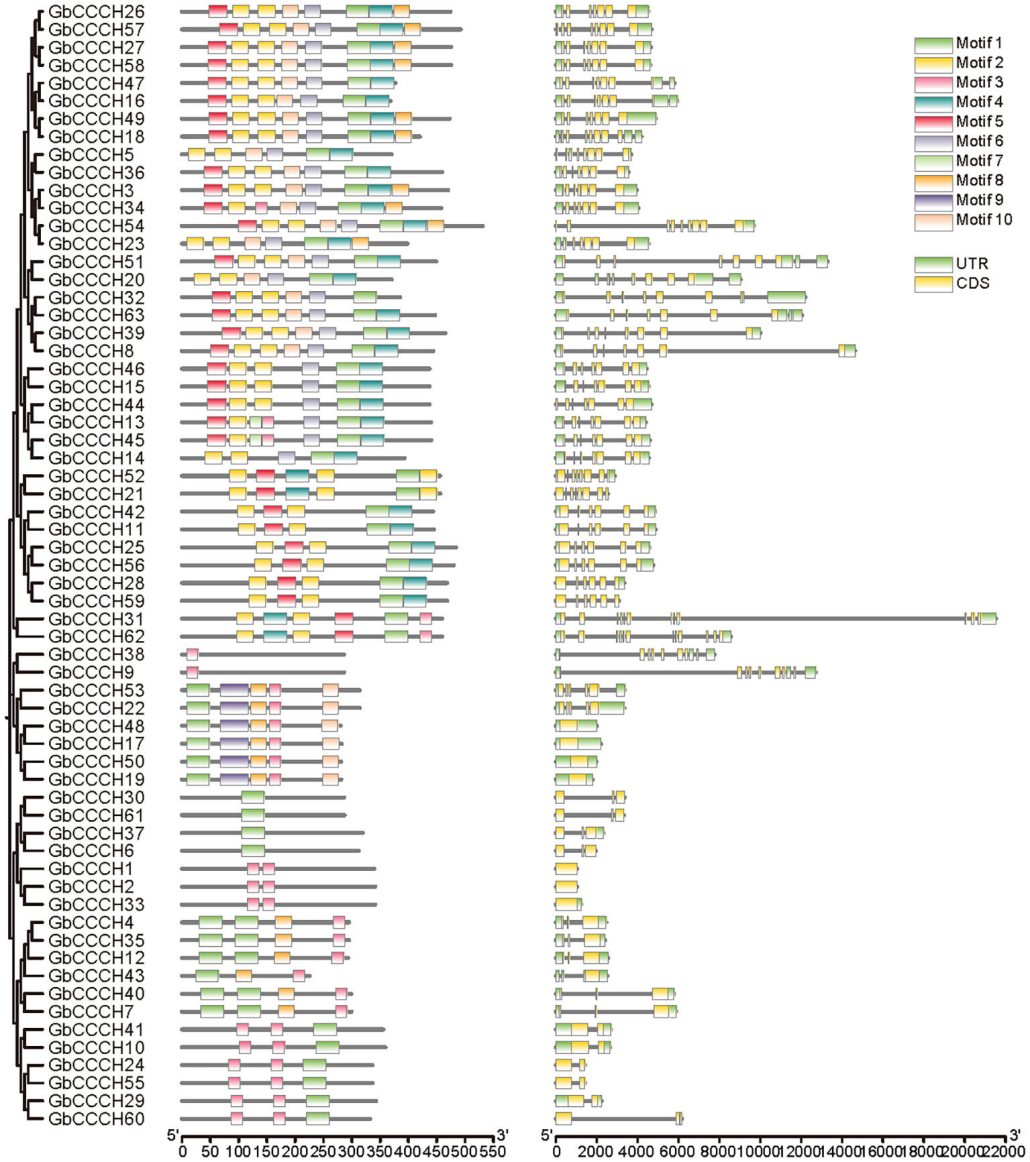
Phylogenetic relationship, motif and gene structures of *CCCHs* in *G. barbadense*. From left to right are evolutionary relationship, structure of conserved motifs and gene architecture.

**Figure 7:**
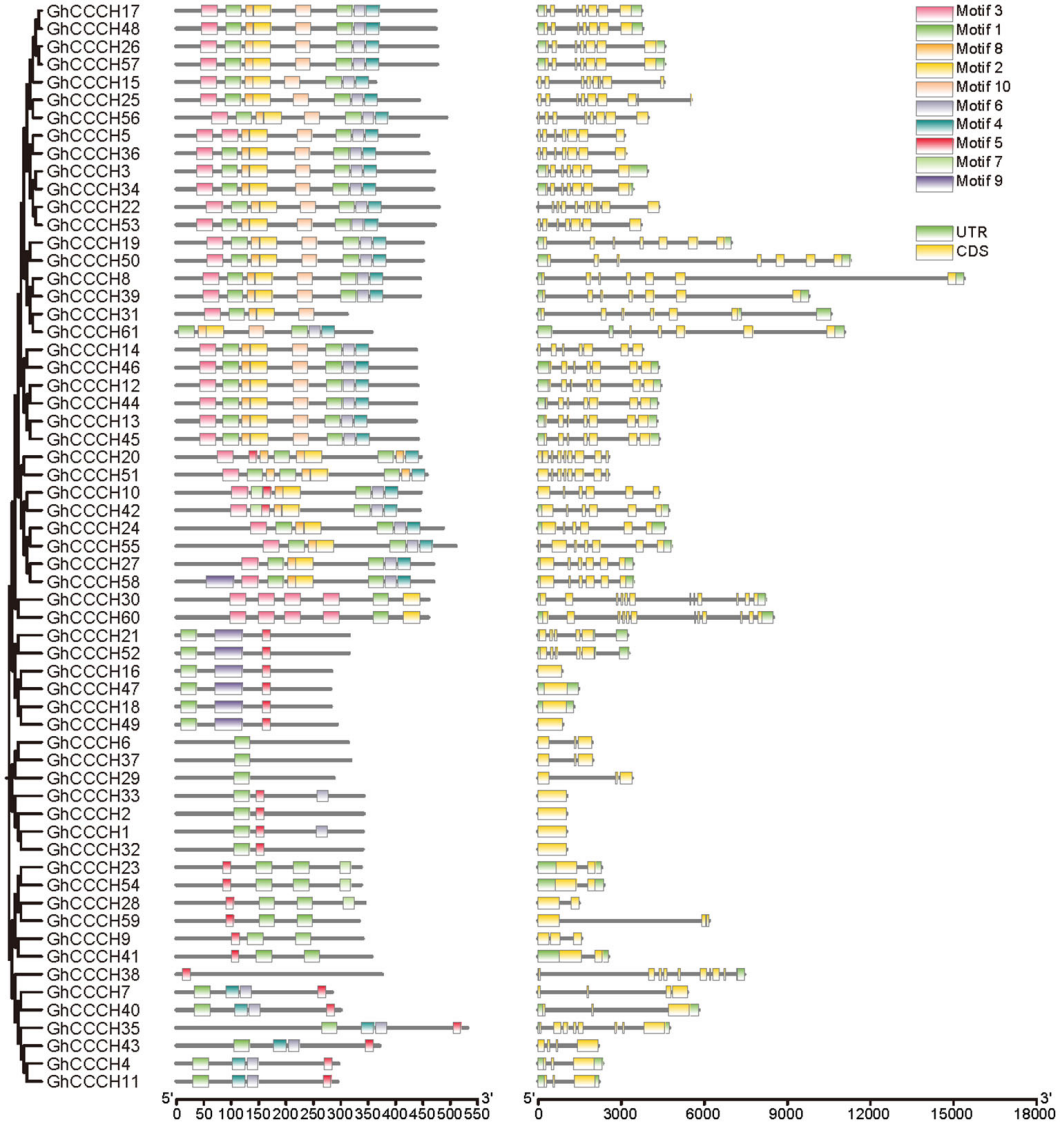
Phylogenetic relationship, motif and gene structures of *CCCHs* in *G. hirsutum*. From left to right are evolutionary relationship, structure of conserved motifs and gene architecture.

To investigate the structural diversity and evolutionary conservation of *CCCH* genes, we systematically analyzed their gene structures and conserved motifs across four cotton species (**Figures 4**–**7**). Gene structure analysis revealed substantial variation in exon number among *CCCH* gene members, ranging from a single exon to more than ten. Overall, genes with more than five exons tended to cluster together, while those with fewer than five exons also formed distinct clusters within each species. Despite the general structural conservation observed within specific phylogenetic clades, certain genes, such as *GaCCCH9*, *GrCCCH17*, *GbCCCH31*, and *GhCCCH38*, exhibited unique exon-intron architectures, suggesting potential functional divergence during evolution.

Conserved motifs were identified using the MEME suite, resulting in the detection of ten distinct motifs (Motif 1-10) across all CCCH proteins. Genes within the same phylogenetic subclade typically shared similar motif compositions and arrangements, reflecting their common evolutionary origin and functional conservation. Moreover, interspecific comparisons revealed that the tetraploid cotton species (*G. hirsutum* and *G. barbadense*) possessed a higher number of conserved motifs per gene compared to the diploid species (*G. arboreum* and *G. raimondii*). Notably, more than half of the *G. raimondii CCCH* genes contained only one or two motifs, indicating possible motif loss or simplification in this lineage. Collectively, these results highlight both the evolutionary conservation and lineage-specific diversification of *CCCH* genes in cotton, providing a foundation for further functional studies.

### *Cis*-element analysis of *CCCH* promoters

**Figure 8:**
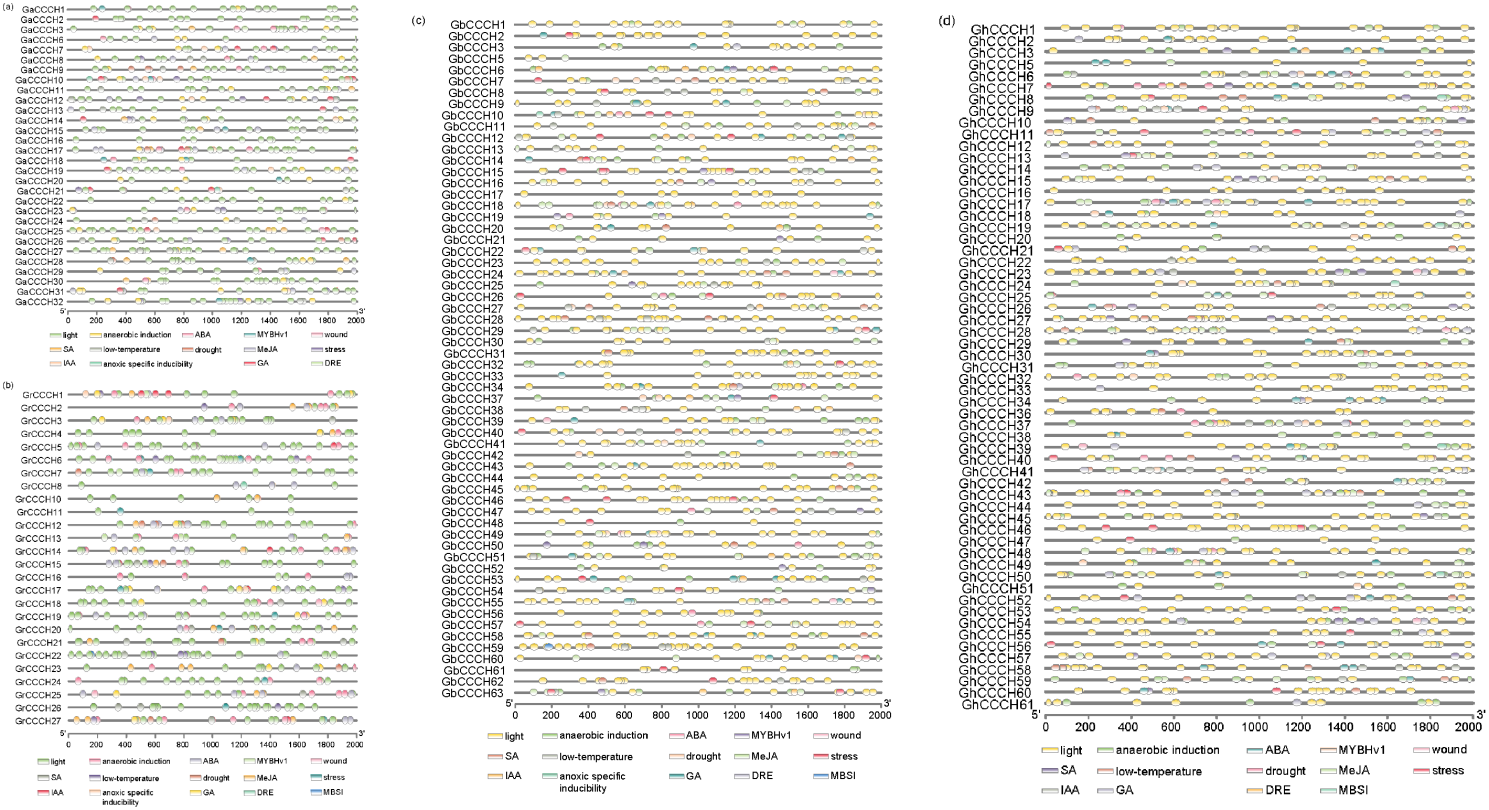
*Cis*-regulatory elements of *CCCH* gene family in *G.arboreum***(a)**, *G*. *raimondii***(b)**, *G*. *barbadense***(c)**, and *G*. *hirsutum***(d)**.

In the analysis of *cis*-regulatory elements in the promoter regions of *CCCH* genes across four cotton species (*G.arboreum*, *G. raimondii*, *G. barbadense*, and *G. hirsutum*), the 2.0-kb upstream sequences were examined using the PlantCARE database. Typical *cis*-regulatory elements including TATA-box and CAAT-box were identified in all species. Beyond these core elements, we detected numerous functional *cis*-regulatory elements associated with light responsiveness, hormone regulation, and stress responses (**Figure 8**). Light-responsive elements were most abundant across all species, suggesting that *CCCH* gene expression may be tightly regulated by photoperiodic and light signaling pathways. Hormone-related elements showed a distinct hierarchical pattern, with methyl jasmonate (MeJA)-responsive elements being the most abundant, followed by abscisic acid (ABA)-responsive elements and then gibberellin (GA)-responsive elements. This consistent ranking of element abundance (MeJA, ABA, GA) was remarkably conserved among all four cotton species examined. Enrichment of MeJA elements (CGTCA/TGACG-motifs) indicates their crucial role in jasmonate signaling, while ABA elements (ABRE) correlate with drought resistance and GA elements (GARE-motif/P-box) may regulate development. Among stress-responsive elements, anaerobic-inducible elements were predominant, followed by widely distributed low-temperature elements (LTR), with smaller quantities of drought-responsive and DRE elements. Additionally, the identification of *MYB*-related elements (MBSI) suggests *CCCH* genes’ involvement in stress responses through *MYB* regulatory networks. These results demonstrate that *CCCH* genes play a key role in environmental adaptation by integrating hormone signaling and stress response pathways.

### Expression profiles of *CCCH* genes in cotton under developmental and abiotic stress conditions

**Figure 9:**
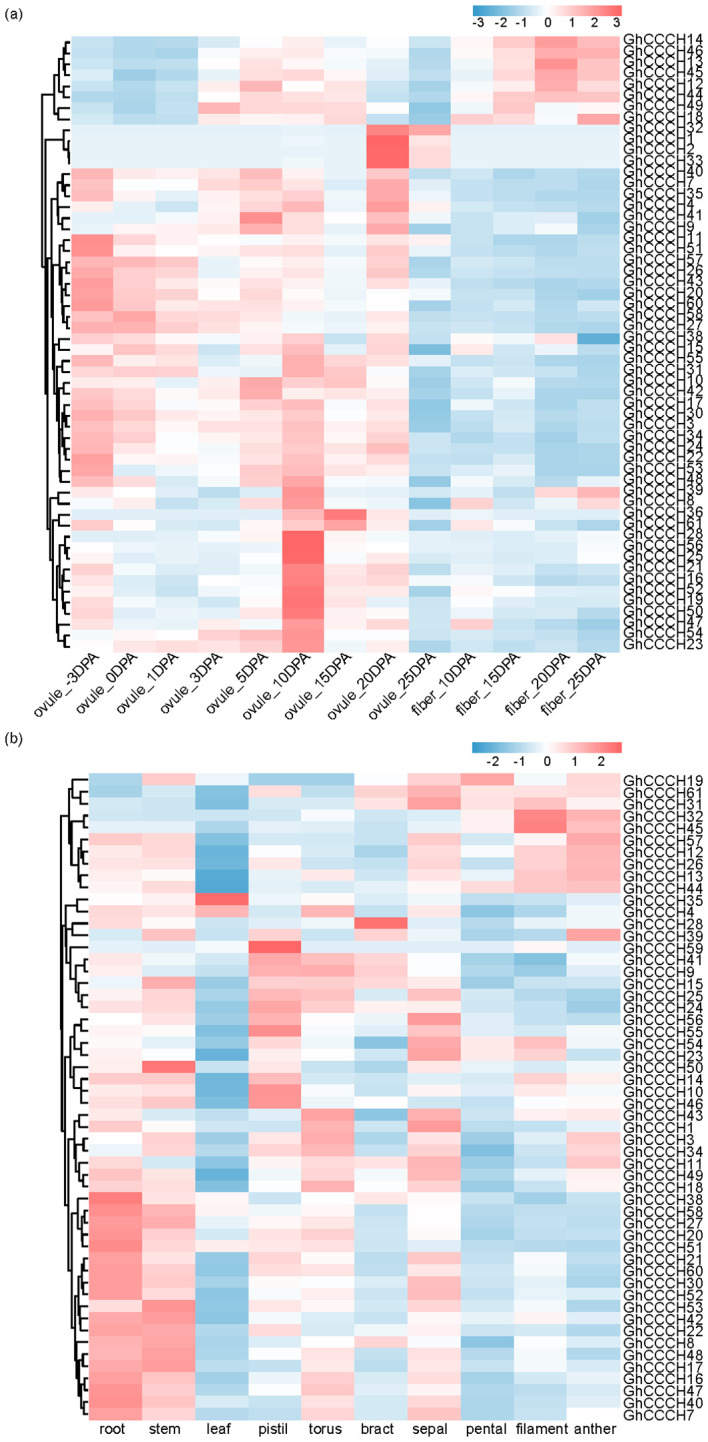
Expression pattern of *CCCH* genes in *G*. *hirsutum* of ovule, fiber **(a)** and various tissues **(b)**.

Integrated expression analysis demonstrated that cotton *CCCH* genes exhibit significant spatiotemporal specificity during developmental processes and abiotic stress responses. As shown in **Figure 9**, tissue-specific expression profiling revealed distinct organizational preferences among *CCCH* genes: *GhCCCH24* and *GhCCCH34* were predominantly accumulated in ovules, whereas *GhCCCH13*, *GhCCCH14* and *GhCCCH46* exhibited preferential expression during fiber development. This complementary expression pattern suggests specialized functions in reproductive tissues and fiber morphogenesis. Additionally, certain genes showed dominant expression in stems, leaves, pistils, and floral organs, indicating their potential roles in organ differentiation and reproductive development. Notably, some genes were ubiquitously expressed across multiple tissues, likely participating in fundamental cellular processes, while others displayed highly restricted expression patterns, implying specialized physiological functions.

**Figure 10:**
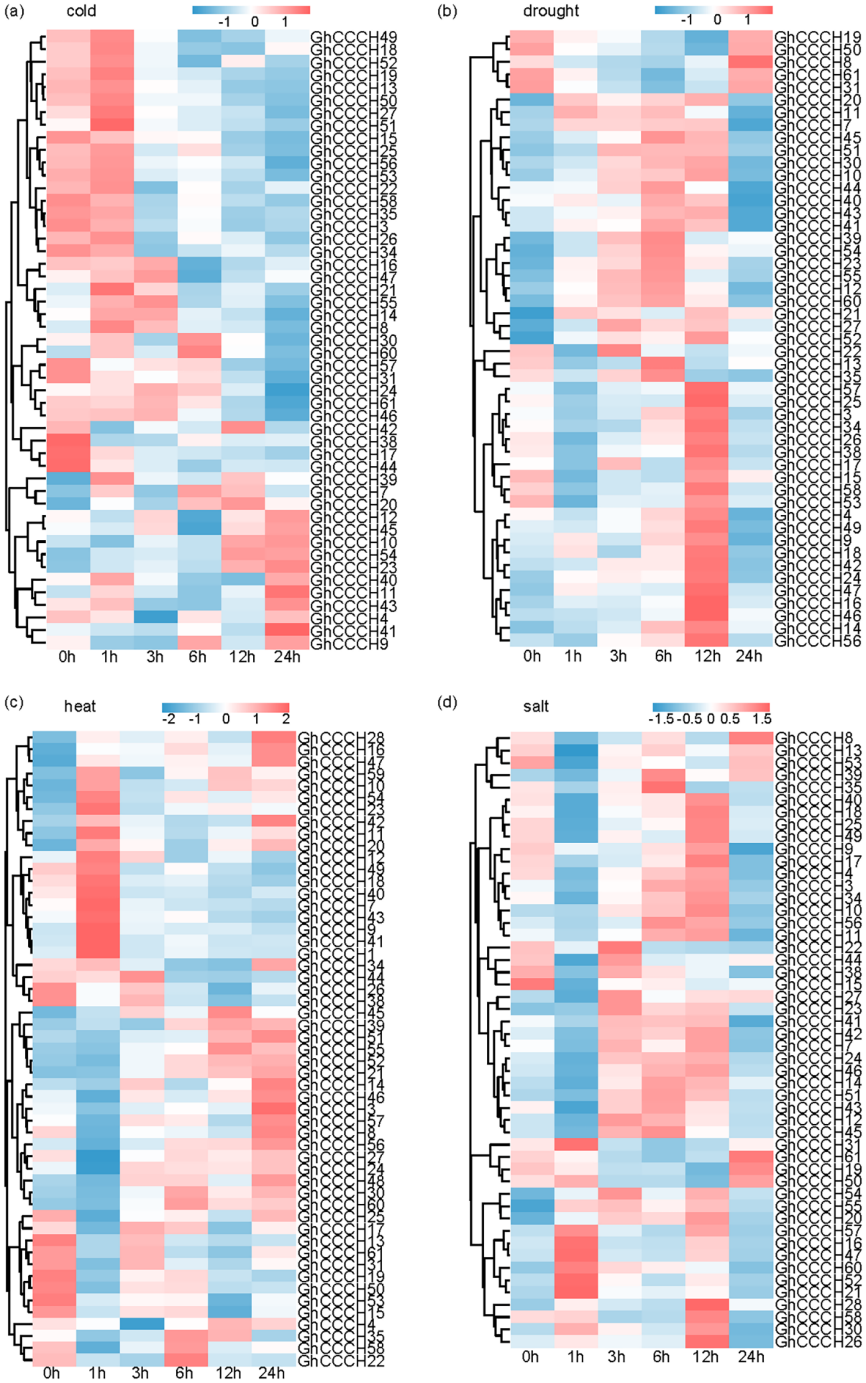
Expression pattern of *CCCH* genes in *G. hirsutum* under different stress treatments: **(a)** cold, **(b)** drought, **(c)** heat, and **(d)** salt. The heatmaps show gene expression levels at 0, 1, 3, 6, 12, and 24 h. Red indicates upregulation, and blue indicates downregulation.

Under abiotic stress conditions (**Figure 10**), *CCCH* genes exhibited dynamic and stress-specific expression patterns. Intriguingly, *GhCCCH13*, which is highly expressed in fibers, was significantly downregulated under cold stress, showing a 2-fold decrease after 24 h of treatment. This contrasting expression pattern may reflect a regulatory balance between fiber development and stress responses. Temporal expression analysis further revealed phased response mechanisms among different genes. *GhCCCH8* was rapidly induced within 1 h of cold treatment, showing a twofold increase relative to the control. *GhCCCH39* and *GhCCCH55* were upregulated 2.9- and 2.1-fold, respectively, within 3–6 h of drought treatment. In contrast, *GhCCCH10* exhibited a 2.1-fold increase after 12 h of salt treatment. *GhCCCH23* showed delayed induction under cold stress, increasing 3.2-fold after 12–24 h. Similarly, *GhCCCH51* and *GhCCCH52* were upregulated (approximately 2.9- and 2.3-fold, respectively) after 12–24 h of heat stress. These expression patterns may reflect a hierarchical response mechanism involved in stress perception and adaptation.

**Figure 11:**
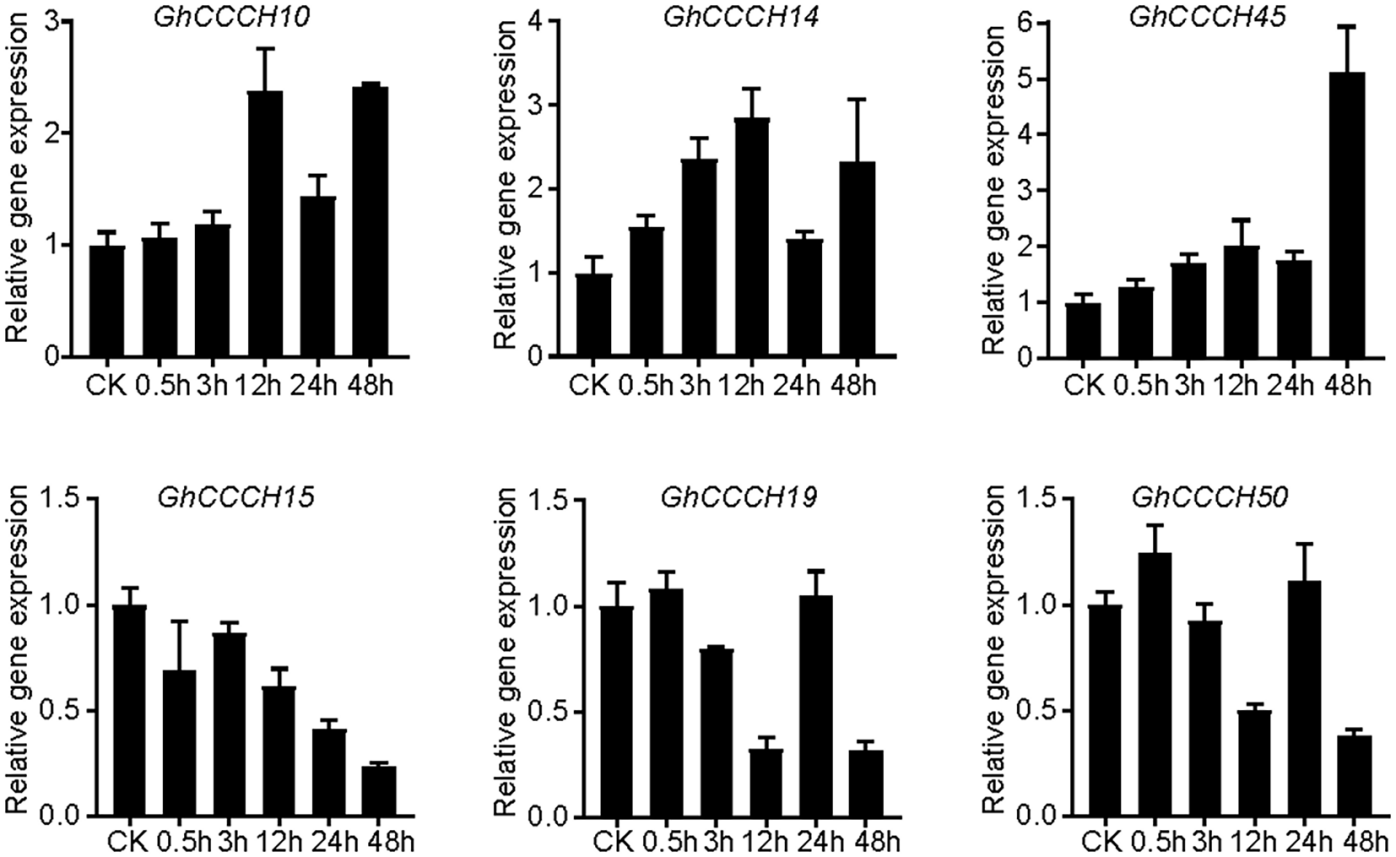
qRT-PCR validation of potential salt-responsive *CCCH* genes at different time points under salt stress.

To further validate the expression patterns of stress-responsive *CCCH* genes observed in the transcriptome analysis, qRT-PCR was conducted using total RNA extracted from *G. hirsutum* cv. ZJ0102 seedlings subjected to salt stress for five time points (0.5, 3, 12, 24, and 48 h) (**Figure 11**). The results showed that the transcript levels of *GhCCCH10*, *GhCCCH14*, and *GhCCCH45* were significantly upregulated after 12h and 48 h of salt treatment, reaching more than twice those of the control (CK). Moreover, *GhCCCH14* expression was markedly induced as early as 3h after salt exposure. In contrast, the expression of *GhCCCH15*, *GhCCCH19*, and *GhCCCH50* was significantly downregulated under prolonged salt stress, with transcript levels decreasing to nearly one-third of the control level. These results were consistent with the transcriptome data, further supporting the reliability of these *CCCH* genes as stress-responsive candidates.

Collectively, these findings systematically elucidate the functional diversification of cotton *CCCH* genes, with distinct members (e.g., ovule-specific *GhCCCH24* and fiber-enriched *GhCCCH14*) governing tissue development while others mediate stress adaptation. The stress-specific induction or suppression of particular genes, coupled with their tissue-preferential accumulation, provides additional insights into the coordinated regulatory networks underlying cotton development and stress resilience.

## Discussion

Zinc finger proteins, particularly CCCH-type members, are key regulators of plant development and stress responses (Kong et al., 2006; Guo et al., 2009; Han et al., 2014; Zhou et al., 2014; Seok et al., 2016; Chen et al., 2020b). In this study, we identified 183 *CCCH* genes across four cotton species and systematically characterized their evolutionary relationships, gene structures, conserved motifs, promoter elements, and expression patterns, providing new insights into how gene duplication, structural diversification, and expression dynamics shape cotton growth and stress adaptation.

Phylogenetic analysis divided cotton CCCH proteins into eight subgroups (**Figure 1**). Tetraploid cotton species (*G. hirsutum* and *G. barbadense*) contained more *CCCH* genes than their diploid progenitors, reflecting whole-genome duplication during polyploidization (**Figure 2**). This expansion parallels other cotton gene families, such as *HSF* and *NCED*, where duplication contributes to regulatory complexity and stress resilience (Rehman et al., 2021; Pei et al., 2021).

Collinearity analysis indicated that most *G. hirsutum CCCH* genes were retained from diploid ancestors, with 51 of 61 genes displaying clear collinear relationships (**Figure 3**). Two *G. hirsutum*-specific genes, *GhCCCH21* and *GhCCCH47*, absent in *G. barbadense*, *G. arboreum*, and *G. raimondii*, likely originated through lineage-specific duplication or neofunctionalization. Drawing on evidence from barley and soybean, where *CCCH* genes show copy-number variation and expression divergence under environmental or domestication pressures, we propose that these two genes represent cotton-specific evolutionary innovations that may contribute to fiber development or stress adaptation (Ai et al., 2022; Hu and Zuo, 2021). Functional validation is needed to confirm their precise roles.

Structural and motif analyses revealed both conservation and divergence within the *CCCH* family (**Figures 4**-**7**). Genes within the same phylogenetic clade generally maintained similar exon-intron organization, suggesting retention of ancestral functional modules. Nevertheless, variations among clades indicate that structural rearrangements may have facilitated functional specialization. For instance, *GhCCCH46*, preferentially expressed in fibers, has a more complex exon-intron structure than stress-responsive genes such as *GhCCCH23*, implying distinct regulatory mechanisms. Motif analysis identified ten conserved motifs, with Motifs 1 and 2 present across all cotton species, reflecting their role in zinc finger stability and RNA-binding activity (**Figures 4**-**7**). By contrast, *G. raimondii* genes exhibited fewer motifs, suggesting potential streamlining during diploid evolution, which could influence binding specificity. This modular diversity is reminiscent of other RNA-binding protein families, such as Pumilio and KH-domain proteins, where motif variation enhances functional versatility (Ray et al., 2013).

Promoter analysis revealed multiple *cis*-regulatory elements associated with abiotic stresses and hormonal responses, including ABRE (abscisic acid), CGTCA/TGACG (jasmonate), and LTR (low temperature) (**Figure 8**). The widespread presence of ABRE motifs suggests ABA-mediated regulation under drought, salinity, and cold stress, consistent with *GhC3H20* function (Zhang et al., 2023). Frequent CGTCA/TGACG motifs indicate potential involvement in MeJA-mediated responses, while the presence of MYB-binding sites in *CCCH* gene promoters implies possible regulation by MYB transcription factors, which are known to play central roles in coordinating abiotic stress responses and secondary-metabolite biosynthesis (Balhara et al., 2024). Auxin- and gibberellin-related elements were also detected, although their functional relevance remains to be validated. These observations provide a mechanistic framework for the stress- and hormone-responsive expression patterns detected in cotton *CCCH* genes.

Spatiotemporal expression profiling revealed clear functional diversification. For example, *GhCCCH24* and *GhCCCH34* were predominantly expressed in ovules, suggesting roles in reproductive development (**Figure 9**), whereas *GhCCCH13*, *GhCCCH14* and *GhCCCH46* were specifically expressed in developing fibers, implicating them in cell elongation and cell wall remodeling critical for fiber quality and yield. Under abiotic stresses (cold, drought, salt, and heat), *CCCH* genes displayed three main expression trends: (1) rapid induction within 1–3 hours (e.g., *GhCCCH8*), indicating early involvement in stress perception; (2) delayed upregulation at 12–24 hours (e.g., *GhCCCH51*), reflecting roles in sustained stress responses or recovery; and (3) repression under stress (e.g., *GhCCCH13*), possibly representing a regulatory balance between growth and defense (**Figure 10**). These distinct temporal patterns highlight that different *CCCH* genes are engaged at specific stages of stress response, coordinating immediate and long-term adaptation. Furthermore, qRT-PCR validation of six salt stress-responsive genes confirmed the reliability of the RNA-seq expression profiles (**Figure 11**). The expression dynamics observed in this study provide direct evidence for the functional specialization of cotton *CCCH* genes under both developmental and environmental contexts.

Collectively, these results highlight the contribution of *CCCH* zinc finger genes to cotton development and stress resilience. Their expansion through polyploidization, structural diversification, and promoter evolution enables responsiveness to environmental cues. Future studies should identify downstream targets, verify MYB-mediated regulation, and perform functional analyses using CRISPR/Cas9 or transgenic approaches. Integrating transcriptomic, proteomic, and metabolomic data under stress and developmental conditions will further clarify the systems-level roles of *CCCH* genes in cotton growth and adaptation.

## Conclusion

In this study, we present a comprehensive genome-wide analysis of *CCCH*-type zinc finger genes in cotton, revealing species-specific members and substantial expression diversity that likely underlie functional specialization in development and stress responses. Our results suggest that polyploidization-driven expansion has shaped the *CCCH* gene repertoire in tetraploid cotton, enhancing regulatory complexity. The identification of stress-responsive genes, including *GhCCCH23*, *GhCCCH51*, and *GhCCCH55*, highlights promising targets for improving abiotic stress tolerance through molecular breeding. Overall, this work advances our understanding of the evolutionary dynamics and regulatory potential of *CCCH* genes and provides a valuable resource for genetic improvement of stress resilience in cotton.

## Data Availability

The original contributions presented in the study are included in the article/**Supplementary Material**. Further inquiries can be directed to the corresponding author.
